# Work profiles of older employees in Germany-results from the lidA-cohort study

**DOI:** 10.1186/s12889-020-09542-3

**Published:** 2020-09-25

**Authors:** Hans Martin Hasselhorn, Michael Stiller, Jean-Baptist du Prel, Melanie Ebener

**Affiliations:** Department of Occupational Health Science, University of Wuppertal, Gaußstraße 20, 42119 Wuppertal, Germany

**Keywords:** Latent profile analysis, Older worker, Work exposure, Working conditions, Health, Work ability

## Abstract

**Background:**

This study investigates whether a typology of work exposure can be established among older workers in Germany. Work exposure comprises physical work, working time quality, work intensity, skills & discretion, social environment, leadership, continued education, earnings and work prospects.

**Methods:**

Latent profile analysis was conducted on a representative sample of the socially insured workforce in Germany born in 1959 or 1965 (*N* = 6277). Seven year-prospective associations between the typology and work-related outcomes (physical and mental health, work ability and work-privacy-conflict) were investigated to establish the distinctness of the profiles.

**Results:**

Five profiles were identified: “Poor Quality” (19%), “Relaxed Manuals” (30%), “Strained non-Manuals” (16%), “Smooth Running” (33%) and “High Flying” (3%). These profiles exhibited diverging patterns of association with the selected outcomes, thus representing qualitatively distinct subgroups of older workers in Germany.

**Conclusions:**

We conclude that a typological approach may broaden the understanding of the ageing work force and the complex interplay of the overall work situation with outcomes of high individual and social relevance such as health, work ability and employment. The five work profiles identified in this study may constitute crucial clusters needed to reliably mirror today’s over-all work exposure patterns in the older work force in Germany. They may allow for the comprehensible monitoring of quality of work and personal life among the older work force during their last working years and their transition to retirement in current times of extending working lives.

## Background

In 2008, 28% of the working population in Germany were aged 50 years or more, ten years later, the proportion has increased to 40% [1]. This trend has not reached its climax yet, as German policy is concerned to keep older workers as long as possible in the labour market [2]. To evaluate the risks and potentials of extended working lives in the older work force, it is of relevance to know and understand this heterogeneous group in its over-all working situation. The aim of this exploratory study is to identify an empirically derived typology of older workers in Germany according to work exposure.

A common theoretical assumption is that during working life, occupational work factors are exerting a chronic impact on the individual workers’ resources such as health [3], attitudes and behaviour [4]. Classical social and occupational epidemiology usually considers single or a combination of specific occupational work factors when investigating occupational risks. Basic research questions resulting from such an analysis approach are, for example, the investigation of the impact of work-organisational factors on work ability [5] and of work stress [6], shift work or extended working hours [7] on health. Research based on the view that a specific work factor – measured by a variable – has an impact on a worker’s work ability or health follows a *variable-centred approach* [8–15]. However, this view does not take into account the relation and combined impact of the wealth of simultaneous occupational work factors in real working life.

Theoretically, any combination of work factors can be found in the work force. Yet, certain typical constellations may preponderate in a work force, thereby characterising groups of distinct occupational exposure. In recent years, occupational epidemiology has increasingly applied this holistic view by employing a so-called *person-centred approach* in analysis [8–15]. Here, not a specific occupational exposure, but the workers in their characteristic and holistic occupational situation are in the focus of interest [15].

Both, the variable- and the person-centred approaches have their strengths and indications for addressing specific research questions. However, only the person-centred approach allows an assessment of the over-all occupational exposure and thus, the description of typical exposure constellations and their respective prevalence in the working population. Which typical combinations of work factors exist in a population? How many workers in a population are working under “good” working conditions? How many are exposed to “poor work”? This type of knowledge is a precondition for portraying the work force in a population with respect to their everyday work situation. It also allows for the observation of typical work groups over time, for example the older work force during their life-course transition from work to retirement.

### Recent attempts in clustering the work force

Over the past decade, in studies employing person-centred approaches, finite mixture modelling has become increasingly popular in occupational epidemiology [e.g. [8–15]. Some of the studies focussed on selected work aspects, such as employment quality [10–12] or psychosocial working conditions [8] including resources [9]. Three studies targeted a broader range of work exposures, namely physical, psychosocial and work organisational factors. Lowe [15] has by means of latent class analysis (LCA) identified six job quality clusters among a representative sample of about 2000 employed workers in Canada, based on 15 job quality items (see Table 1). In a similar approach, Vanroelen et al. [13] identified five profiles among about 10,000 employees in the Flemish part of Belgium. Finally, the EU-agency „Eurofound“ (European Foundation for the Improvement of the Working and Living Conditions) has identified five distinct groups of workers with characteristic work profiles using data from the 2015 round of the European Working Conditions Survey [14]. This survey covers roughly 27,000 workers from 28 European countries (cf. Table 1). In contrast to the earlier studies, Eurofound has used metric work quality indices for identifying distinct classes by applying latent profile analysis (LPA).

**Table 1:** Overview of work profiles in LCA studies assessing the broad range of work exposures

Profile (% in sample)	Profile description
Lowe [15], 6 profile solution, 2002 workers in Canada	
1. “total rewards” (6%)	non-manual work, most positive scorings for all work indicators assessed, except pay and benefits (ranking 2nd)
2. “decide and say” (9%)	manual work, good scorings for skills and discretion, but poor on pay and work life balance
3. “relationships and balance” (20%)	mixed physical/non-physical work group, good scorings for social work environment indicators, but poor on discretion
4. “economics and support” (12%)	non manual work, positive scorings for all work indicators except discretion, best scorings for pay and benefits
5. “security” (16%)	mostly manual work, good benefits, rather healthy, safe and secure work place, otherwise low scorings, including poor social work environment
6. “few rewards” (37%)	all manual work, most negative scorings for all work indicators assessed
Vanroelen [13], 5 profile solution, 10,074 workers in the Flemish part of Belgium	
1. “low stress” (26%)	predominantly non-manual work, low demands, high control, good social relations, high job security
2. “passive manual” (24%)	manual work, average demands, low control, average level social relations
3. “human contacts” (21%)	moderate physical demands and comparably adverse exposure for social work environment indicators
4. “high demand” (18%)	non-manual work, high work demands, high control and an advantageous social work environment, high job security
5. “high stress” (11%)	very high quantitative and physical work demands, adverse social environment, atypical work schedules, high job insecurity
Eurofound [14], 5 profile solution, 26,648 workers from 28 European countries	
1. “high flying” (21%)	non-manual profile, high on skills and discretion, good social environment, good earnings and prospect, higher than average work intensity
2. “smooth running” (25%)	non-manual profile, very good social environment and working time quality, very low work intensity. However, low on skills and discretion and especially earnings
3. “active manual” (21%)	distinctly manual profile with good social environment and prospects, otherwise poor working time quality and work intensity
4. “under pressure” (13%)	a mixed, manual non-manual profile characterised by least favourable scores for the social work environment and work intensity. Else higher skills and discretion scores and better earnings, but poor working time quality
5. “poor quality” (20%)	mostly manual profile with less favourable scorings for all indices. Distinctly low skills and discretion, prospects and earnings.

The overview of the three work quality typologies in Table 1 indicates that solutions with five to six profiles may be expected when clustering the work force. In each study, a least favourable profile can be identified scoring worst on almost all work indices included in the analyses, typically a manual work profile. In contrast, the identification of a most favourable (typically non-manual) profile is not as clear-cut: a *low stress* profile presented by Vanroelen et al. [13] ranks first for many, but not all work indices. In the Eurofound [14] analysis it remains open whether work exposure of the *high flying* or the *smooth running* is to be preferred. Overall, the results of all studies underline the multidimensionality of work itself and at the same time a certain homogeneity of the working population on a higher level with respect to work exposure.

### Purpose of the present study

So far, no specific work profiles of the German working population have been published. From a national perspective, a shortcoming of the Eurofound profiles is that they reflect the work exposure of the combined work force of 28 European countries. However, there are substantial differences of profile distributions between countries [cf. 14, also 11], indicating that different profiles may emerge when only single countries are investigated. Furthermore, the Eurofound analyses comprise the work population covering the full range of working age [14]. However, for certain scientific and socio-political issues it may be advantageous to focus on specific age groups. For example, when the interest lies in the understanding of the work force transiting from work to retirement, the aim should be to capture the specific work situation of the older work force, which is a function of decades of work-related transitions, experiences, trajectories and contexts [3]. Finally, the cross-sectional data of the European Working Conditions Survey do not allow for longitudinal observations and therefore inferences on outcomes over time.

Therefore, the main aim of this contribution is to identify characteristic work exposure profiles in the older socially insured working population in Germany by means of a person-centred analysis approach [16]. The identified profiles will be described with respect to socio-demographic characteristics of the workers assigned to each of them. Finally, it will be investigated, how the groups differ cross-sectionally and develop over seven years with respect to work-related outcomes.

## Methods

To investigate this, a LPA was conducted on cross-sectional data (wave 1) and the identified profiles were related to four outcomes based on measurements from three waves (1 to 3), thereby constituting a hybrid cross-sectional and longitudinal analysis.

### Study design, sample selection and description

Data of the German lidA Cohort Study on Work, Age, Health and Work Participation was used (www.lida-studie.de). lidA is representative for employed people subject to social security contributions (no self-employed or sworn civil servants), born in either 1959 or 1965 and thus for marking cohorts of the German “baby boomer” generation. Participants were interviewed at home with respect to their work, health, personal life and employment participation, by means of computer-assisted personal interviews. So far, interviews have been conducted at three time points: in 2011 (wave 1) 6585 people participated, in 2014 (wave 2) 4244 and in 2018 (wave 3) 3586. The response rate at wave 1 was 27.3% (RR5 according to AAPOR [17], counting complete cases only) and the cooperation rate was 32.6% (COOP3 according to AAPOR [17], excluding those unable to do an interview from the base) which is similar to that of other German surveys of comparable study design [e.g. [18, 19]. In all instances, the samples are representative for the socially insured working population of the respective age cohort in 2009, when the sample was randomly drawn [20–22]. A detailed description of the lidA Cohort Study and its design is found elsewhere [23].

The sample for the LPA comprised of 6277 participants of the first wave of the lidA study, all being employed workers for at least one hour per week. The number of workers born in 1959 was 2752 (43.8%) and those born in 1965 3525 (56.2%). This uneven age distribution reflects deliberate oversampling of the younger cohort to compensate for future loss due to attrition [23]. Two thousand four hundred ninety-seven participants were male (46.9%) and 3330 were female (53.1%).

To be able to follow up profile-related differences of the outcomes over time, participants with valid data for all three waves were selected (*n* = 3118). In this sample, the proportion of women was 55.1% (*n* = 1718) and significantly higher than in the LPA-sample (χ^2^_(1)_ = 5.01, *p* = .025). Further, the proportion of those born in 1959 was significantly higher in the sample for longitudinal analysis (45.9%; *n* = 1430; χ_(1)_ = 5.39, *p* = .020).

### Calculation of work indices

For the determination of the work profiles, a procedure was adopted as conducted by Eurofound with data from the „6th European Working Conditions Survey “[14]. In a first step, the seven work exposure indices used by Eurofound were re-constructed with variables available in the lidA-study. Two further indices, “continued education” and “quality of leadership”, were added because they were considered to represent distinct work exposures in German working life. The resulting nine indices are supposed to represent the totality of resources and demands of work exposure and are displayed in Table 2. Apart from using single-item measures such as shift work or the amount of working time being exposed to adverse physical exposure, selected scales derived from the „Copenhagen Psychosocial Questionnaire “[COPSOQ [24]; and items of the „Effort-Reward-Imbalance Questionnaire “[ER I[25]; were used for index construction. The final nine indices correlated in most instances with *r* < │.10│ and always below │.50│.

**Table 2:** Composition of indices, weighting of contents and descriptive statistics

Index	Scales/ items included (weighting factor)	Descriptives
Physical exposure	Proportion of working time: sitting (1); bending, kneeling/ lying/working above head (1); lifting heavy loads (1); repetitive movements (1)	50.0 (50.0)^a^
Working time quality	Shift work (1); night shifts per month (1); having another job (1)	100.0 (33.3)^a^
Work intensity	COPSOQ-Scale „quantitative demands – 4 items “(2); frequency of interruptions at work (1)	47.6 (25.5)^b^
Skills & discretion	COPSOQ-Scale „influence at work – 3 items “(1); COPSOQ-Scale „possibilities for development – 3 items “(1)	50.8 (21.6)^b^
Social environment	COPSOQ-Scale „support from colleagues – 2 items “(1); ERI item: receiving acknowledgment from colleagues (1)	69.7 (29.0)^b^
Quality of leadership	COPSOQ-Scale „quality of leadership – 3 items “(3); ERI-item stress due to lack of recognition from superior (1)	61.1 (22.2)^b^
Continued education	Participation in continued education in the last 12 months: 0 = no, 1 = yes	.20^c^
Earnings	Monthly equivalised household net income	16.2 (7.5)^b^
Prospects	Open ended or fixed-term work contract (1); recent job cuts/dismissals in enterprise (1); risk of losing one’s job (1); promotion prospects (1); chances for occupational advancement (1)	60.0 (20.0)^a^

*Note.* a = median and interquartile range are displayed for ordinal indices; b = mean and standard deviation are displayed for metric indices; c = proportion in the sample

In order to calculate a score per index, at least half of the respective items and scales had to have valid values. After weighting with the factors indicated in Table 2, all raw scores for the items and scales contributing to one index were summed up. Finally, the sum scores were transformed to an index ranging from 0 (adverse exposure) to 100 (favourable exposure). The earnings index was calculated as the monthly net equivalised household income according to OECD [26]. Regarding scale type, ordinality was assumed in the subsequent analyses for “physical exposure”, “working time quality” and “prospects”, while “continuous education” was treated as nominal and the remaining indices as metric. The proportion of missing values was below 1.9% for eight indices. However, for income it was 19.9% which corresponds to the mean refusal rate in Europe to disclose earnings [14]. As a consequence, missing values were replaced by means of stochastic regression imputation [27] to handle systematic selection and to assure a high number of participants.

### Work-related outcomes

Posterior profile membership was linked to selected, commonly used work-related outcome measures over time to demonstrate distinctness and thus validity of the latent profiles identified in the prior analysis steps. This procedure was done analogous to “step 3 analysis” in LPA [28]. Here, physical and mental health, work ability and work-privacy-conflict were chosen as so-called external outcomes to conceptually reflect this procedure as done by Eurofound [14]. Physical and mental health were assessed by the respective SF-12 short scales, the physical component summary and the mental component summary as described by Nübling et al. [29]. Work-privacy-conflict was measured by a 2-item COPSOQ scale covering both the mental strain as well as the time strain component of a conflict between work and private life [30]. The second dimension of the Work Ability Index [31] was used as a measure for work ability. This two-item scale, ranging from 2 (=no work ability) to 10 (=optimal work ability), assesses the estimation of one’s own mental and physical work ability. It has been validated and recommended recently for the use in epidemiological studies [32].

### Statistical analyses

In a first step, a „latent profile analysis “[33] was conducted in LatentGOLD 5.1 [Statistical Innovations, Belmont MA, USA] to identify different patterns of work exposure among subjects on a latent variable that comprises *k* classes (or in our case: profiles). In classical LPA, all constituting indicators are metric, yet in the present study the scale type was mixed as outlined above and has been modelled accordingly in the final analyses. The LPA is model-based and assumes that associations between the observed indicators can be explained by a latent variable with *k* profiles comprising different manifestations of the indicators chosen [16]. After model estimation, classification of a heterogeneous sample into homogeneous subgroups (the *k* profiles) is possible by means of their respective posterior profile membership probabilities, and thus unobserved heterogeneity in the sample can be described [28]. Model selection was based on three premises: Formal criteria for model selection were the Akaike- (AIC) and the Bayesian information criterion (BIC), where lower values indicate better model fit [28]. Secondly, parsimonious solutions were favoured with profiles sufficiently differentiating among employees. Finally, a plausible interpretability guided the selection of the optimal number of profiles [16, 34]. Data preparation and subsequent analyses were done with IBM SPSS Statistics 25. These included χ^2^-tests to examine the demographic distribution across profiles. Correlation analysis confirmed that the nine single indices correlated differently and in varying amounts with the health components, work-privacy-conflict or work ability. In almost all instances, the correlation coefficients were significant (*p* < .001) ranging from small to medium size. This correlational pattern prompted further investigation of differences between profiles in all instances. Mixed-design ANOVA’s were performed to test for mean differences between the profiles on the outcomes across three time points. Beforehand, inspection of the outcome distributions yielded skewness values ranging from − 0.70 to 0.43, and kurtosis values ranging from 2.42 to 3.48, thus indicating acceptable distributional properties for further analyses. The longitudinal analyses were done for those with valid data for all three time points. This way, within and between group effects can be simultaneously identified without having to consider attrition and health-related selection effects. Greenhouse-Geisser corrections were used because sphericity was violated for all work-related outcomes except for mental health. Post-hoc comparisons were Bonferroni-adjusted to a significance level of .05. Effect sizes for main and interaction effects with η^2^ ≥ .01 were classified as small, η^2^ ≥ .06 as medium and η^2^ ≥ .14 as large [35].

## Results

### Determination and interpretation of profiles

The LPA was conducted with an increasing number of profiles. The AIC and BIC did not reach a minimum up to a solution with 10 profiles. As both criteria dropped in smaller steps for solutions with more than five profiles, this solution was favoured (AIC/BIC – four profiles: 322421.1/323109.2, five profiles: 321545.7/322301.1, six profiles: 320995.8/321818.7 and seven profiles: 320445.6/321335.9). The fifth group in the five-profile solution was extremely small (about 3%). Nonetheless, it was deemed meaningful because it had already emerged in the four-profile solution and remained stable across all further solutions.

**Fig. 1:**
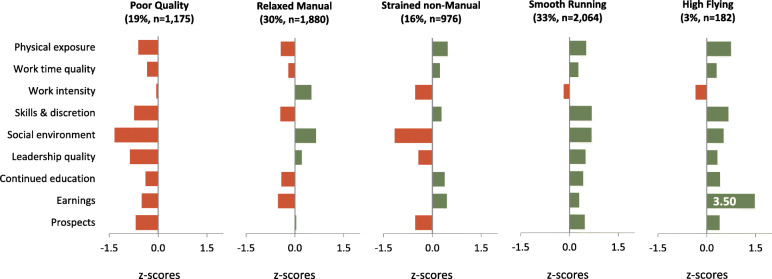
Relative manifestation (z-values) of the indices across profiles; red bars to the left indicate unfavourable manifestation, green bars to the right indicate favourable manifestation; due to space restriction the earnings bar of the High Flying profile was shortened and the actual z-value (3.50) was displayed

In solutions with six or more profiles, they mostly differed by one index only, going along with small, instable profiles and impeding a straightforward interpretation. The stability of the favoured five-profile solution was investigated by recomputing the LPA in three randomly drawn subsamples containing 50% of the original sample each [28]. In the first and third subsample, AIC and BIC did not reach a minimum whereas in the second one, both indicators suggested a nine-profile solution. In all subsamples, the numeric decrease for AIC and BIC was smaller for solutions with more than five profiles and the percentage distribution of employees in the five profile solutions was comparable to that in the full sample analysis, thus indicating relative stability. In the full sample, mean posterior profile probabilities ranged between .84 and .94 across the final five profiles indicating fairly reliable classification [36]. Profile characteristics and group sizes of the final five-profile solution are shown in Fig. 1 and described as follows:

Nearly one of five older employees (18.7%) was assigned to the “Poor Quality” (PQ) profile, a group of predominantly manual workers characterised by unfavourable work exposures for almost all indices. The “Relaxed Manual” (RM, 30.0%) also comprise of manual workers with a profile resembling that of PQ but with favourable manifestations of the indicators for social interaction and with lowest work intensity. The “Strained non-Manuals” (SnM, 15.5%) conceptually depict the complement to the RM profile exhibiting lower-than-average scores for social work environment and prospects while work intensity is clearly highest (red low z-score). The large non-manual group of the “Smooth Running” (SR, 32.9%) is characterised by favourable non-physical work exposures with respect to all indices except work intensity. The profile of the small non-manual group “High Flying” (HF, 2.9%) is almost identical with that of the SR, apart from the extremely high earnings in this subgroup and higher work intensity.

### Demographic characteristics across profiles

The proportion of employees born in 1965 was slightly above 50% across all profiles thus representing the overall distribution in the sample, χ^2^_(4)_ = 2.53, *p* = .639. There were differences for the proportional distribution of sex, educational level [37] and weekly working hours, as well as the requirement level of task complexity and occupational area [38] (cf. Table 3).

**Table 3:** Distribution of older employees across profiles (percentage)

	Poor Quality (*n* = 1175, 18.7%)	Relaxed Manual (*n* = 1880, 30.0%)	Strained non-Manual (*n* = 976, 15.5%)	Smooth Running (*n* = 2064, 32.9%)	High Flying (*n* = 182, 2.9%)
Sex: χ^2^_(4)_ = 14.74, *p* = .005					
Male	48.2	44.1	45.4	48.9	54.4
Female	51.8	55.9	54.6	51.1	45.6
Educational level: χ^2^_(8)_ = 774.95, *p* < .001					
Low	36.3	37.9	13.1	15.7	5.5
Medium	54.7	54.0	55.2	53.3	47.0
High	9.0	8.1	31.6	31.0	47.5
Job task requirement level: χ^2^_(12)_ = 1401.07, *p* < .001					
Un−/Semiskilled task	15.6	16.6	1.9	1.6	a
Skilled task	68.7	70.1	44.4	44.6	> 20.0^a^
Complex task	10.7	8.9	25.7	24.0	23.3
Highly complex task	5.1	4.4	28.0	29.8	50.0
Weekly working hours: χ^2^_(8)_ = 264.54, *p* < .001					
Full-time (> 34 h/week)	69.4	59.6	74.8	74.5	88.8
Part-time (20 to 34 h/week)	19.9	22.4	20.9	19.8	> 5.0^a^
Part-time (< 20 h/week)	10.8	18.0	4.3	5.6	a
Occupational area: χ^2^_(32)_ = 872.79, *p* < .001					
Armed forces personnel	0.0	0.0	0.0	0.0	0.0
Agriculture, forestry, gardening and fishing	2.0	2.0	0.5	1.0	0.0
Production of raw materials and goods, and manufacturing	27.5	24.3	15.3	17.0	9.4
Construction, architecture, surveying, and technical building services	4.5	7.2	3.1	4.8	3.3
Natural sciences, geography, and informatics	1.5	2.0	5.8	5.8	7.2
Traffic, logistics, safety and security	20.6	18.6	4.2	3.9	3.3
Commercial services, trading, sales, hotel business and tourisms	12.0	13.4	8.1	9.6	8.9
Business organisation, accounting, law, and administration	13.5	13.5	30.0	29.3	48.3
Health care, the social sector, teaching and education	16.9	18.1	28.7	25.0	15.0
Philology, literature, humanities, social sciences and economics, media, art, culture and design	1.6	0.9	4.3	3.7	4.4

*Note. N* = 6277; ^a^ Small cell numbers cannot be displayed for reasons of data protection

The proportion of women was above 50% in all profiles except in the HF where the relation was reversed. With respect to education, task complexity level and also occupational area, the distribution was quite similar for two manual profiles PQ and RM, and also for two non-manual profiles SnM and SR. The first predominantly cover participants with low and medium level education, and with low and medium task complexity levels. Occupational areas with higher prevalence in these profiles are agriculture, production, logistics and safety and certain services. The second, SnM and SR, mainly cover the upper range of education and task complexity level in their groups. Full time work is most and part-time work less than 20 h/week is least prevalent. Here, natural and IT sciences, business organisation and administration, the health and social sector and humanities are more prevalent. The HF show the largest proportions of workers with high education and task complexity, while working part time is less frequent. Almost half of all participants assigned to this profile work within business organisation and administration.

### Differences in work-related outcomes

**Fig. 2:**
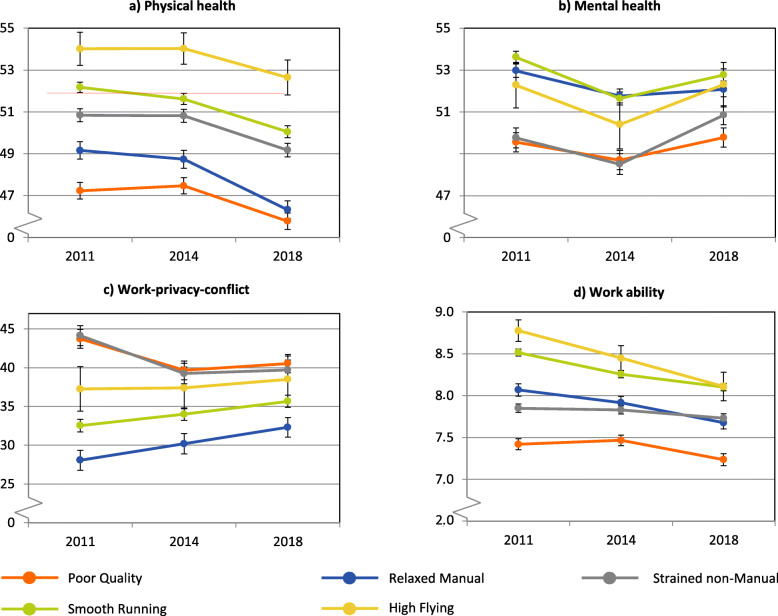
Association of profile membership with work-related outcomes across three time points in seven years (error bars represent standard errors). a) Physical health (higher scores indicate better health). b) Mental health (higher scores indicate better health). c) Work-privacy-conflict (higher scores indicate higher work-family-conflict). d) Work ability (higher scores indicate a higher work ability)

There were substantial group differences in all work-related outcomes investigated and also diverging profile patterns for the time course of the outcome variables over seven years (see Table 4 and Fig. 2). Regarding physical health, profile membership (*F*_(4; 2844)_ = 43.10, *p* < .001, part. η^2^ = .06) and time (*F*_(2; 5658)_ = 42.56, *p* < .001, part. η^2^ = .02) showed a significant main effect. The profile*time interaction was not significant (*F*_(8; 5658)_ = 1.60, *p* = .119, see Fig. 2 a). All profiles differed significantly from each other in all three instances with a plausible gradient “good to poor health” from HF, SR and SnM to the manual profiles RM and PQ. For all profiles, the physical health means did not change significantly between 2011 and 2014 but had decreased by 2018, except for the HF, where the small group size and high dispersion prevented significance of changes over time.

**Table 4:** Descriptive statistics for work-related outcomes by work profile over 7 years

	Physical health	Mental health	Work-privacy-conflict	Work ability
Profile	*M (SD)*	*M (SD)*	*M (SD)*	*M (SD)*
Poor Quality				
*n*	478	478	489	476
2011	47.1 (9.1)	49.6 (10.4)	43.7 (27.5)	7.4 (1.5)
2014	47.4 (8.8)	48.6 (10.5)	39.5 (27.4)	7.5 (1.5)
2018	45.9 (8.7)	49.8 (10.6)	40.1 (27.0)	7.2 (1.7)
Relaxed Manual				
*n*	777	777	784	779
2011	49.3 (8.8)	53.1 (8.9)	27.8 (26.2)	8.1 (1.4)
2014	48.9 (8.9)	51.8 (9.3)	30.2 (26.5)	7.9 (1.4)
2018	46.5 (9.2)	52.0 (9.7)	32.1 (26.7)	7.7 (1.5)
Strained non-Manual				
*n*	473	473	478	474
2011	50.7 (8.7)	49.7 (9.9)	44.0 (27.4)	7.9 (1.6)
2014	50.6 (8.9)	48.7 (10.3)	39.5 (27.3)	7.8 (1.5)
2018	49.8 (9.2)	50.8 (9.3)	40.1 (26.8)	7.7 (1.5)
Smooth Running				
*n*	1031	1031	1051	1039
2011	52.0 (8.1)	53.6 (8.9)	32.6 (25.4)	8.5 (1.3)
2014	51.5 (8.6)	51.7 (10.0)	33.9 (26.0)	8.3 (1.4)
2018	49.9 (9.2)	52.8 (9.4)	35.8 (25.7)	8.1 (1.4)
High Flying				
*n*	90	90	90	88
2011	54.0 (7.4)	52.3 (10.2)	37.6 (27.1)	8.8 (1.2)
2014	54.0 (7.0)	50.4 (11.0)	37.5 (25.3)	8.4 (1.4)
2018	52.7 (7.8)	52.3 (10.0)	38.8 (28.1)	8.1 (1.6)

Also for mental health, significant main effects for profile membership (*F*_(4; 2844)_ = 24.17, *p* < .001, part. η^2^ = .03) as well as time (*F*_(2; 5688)_ = 16.73, *p* < .001, part. η^2^ = .01) were evident. Here, the profile*time interaction was significant (*F*_(8; 5688)_ = 2.44, *p* = .013, part. η^2^ = .00, see Fig. 2 b). However, the effect size did not indicate practical significance. For mental health, all profiles exhibited a dip in the 2014 assessment. In contrast to the findings for physical health, the profiles showed more group overlap with respect to mental health and a diverging gradient: Highest scores were found for the SR, RM and also HF and lowest for SnM and PQ.

With respect to the work-privacy-conflict a significant main effect for profile membership was found (*F*_(4; 2887)_ = 31.88, *p* < .001, part. η^2^ = .04), but not for time (*F*_(2; 5735)_ = 1.69, *p* = .184). However, the profile*time interaction was significant but with low effect size (*F*_(8; 5735)_ = 6.43, *p* < .001, part. η^2^ = .01, see Fig. 2c). The PQ and SnM exhibited the highest work-privacy-conflict in all instances and showed a decrease from 2011 to 2014. In contrast, the SR and especially the RM had the lowest but increasing levels for this outcome between 2011 and 2018. The HF remained relatively stable over time ranking between the higher and lower scoring profiles.

Finally, also for work ability a distinct profile pattern was found. All effects were significant (see Fig. 2 d) – the main effect for profile affiliation (*F*_(4; 2851)_ = 62.06, *p* < .001, part. η^2^ = .08), the main effect over time (*F*_(2; 5630)_ = 36.23, *p* < .001, part. η^2^ = .01) and also the profile*time interaction (*F*_(8; 5629)_ = 320, *p* = .001, part. η^2^ = .00). The profile gradient was widely constant over time with highest work ability scores for HF and SR, followed by RM and SnM, and with low scores at all instances for PQ. Work ability decreased for all profiles over time except among the SnM.

## Discussion

In this study, a typology of contemporary work exposure among older employees in Germany was established, based on recent representative data. The procedure was comparable to the approach performed by Eurofound [14]. The appropriateness of both, the chosen indices and the final profiles, was assessed by means of theoretically and empirically established work-related outcomes. In summary, the findings suggest the existence of five distinct profiles of work exposure among the German baby boomer workforce. This helps scientific and policy stakeholders to better conceive the currently employed baby boomer generation in Germany in its holistic work situation.

### Profile summary

The first profile identified is that of *Poor Quality* (PQ) work. Almost 20% of all workers belong to this profile which is characterised by least favourable exposures for eight of nine work indices. It is dominated by mean low qualification levels and mainly manual jobs and professions. The group exhibits the most adverse scores for all work-related outcomes, be it health, the work-privacy-conflict or work ability.

The *Relaxed Manual* (RM) profile covers 30% of all workers. Like the PQ profile it is characterised by, on average, low qualification levels, mainly manual jobs and professions and several poor work indicators. The differences, however, are the more favourable manifestations of the social work exposure indicators and the lowest work intensity of all profiles. These positive exposures are reflected by better mental health and very low work-privacy-conflict scores. Physical health, however, is rather low.

With respect to the manifestation of the work exposure indices, the *Strained non-Manual* (SnM) constitute the inverse counterpart of the RM with predominantly non-manual work, adverse scorings for social work indicators, work intensity, and also prospects. Sixteen percent of all workers belong to this group, mainly from business administration and organisation, the health and social sector and also scientists. The SnM profile shows adverse mean scores for mental health and work-privacy-conflict, while physical health and work ability are on average level.

The largest profile (33%) is the *Smooth Running* (SR) with an all-favourable work exposure profile opposite to that of PQ, except for above average work intensity. The predominant professions are similar to those of SnM, yet, in contrast to SnM, mental health is highest among SR, work ability rather high and the work-privacy-conflict rather low.

The smallest profile (3%) is that of the *High Flying* (HF), the profile with the most favourable work exposure manifestations except for rather high work intensity. The very high score for earnings is a distinct feature of this profile. Specifically, business organisation and administration, but also scientists are more frequently found in this group. While scores are highest for physical health and work ability, the profile reaches medium group levels for mental health and work-privacy-conflict only.

### Relation to further profiles

There is substantial accordance between the profiles identified here and the profile patterns of the three studies using comparable approaches (cf. Table 1). Not surprisingly, the PQ profile finds equivalents in all studies, the “few rewards” profile in Canada [15], the Flemish “high stress” [13] and the European “poor quality” profiles [14]. The RM profile identified in this study is to a certain degree equivalent to the Canadian “relationships and balance” profile [15], the “passive manual” in Belgium [13] and the “active manual” identified by Eurofound [14]. The SnM profile has an overlap with the “under pressure” profile by Eurofound [14]. The favourable SR profile would match the combination of the profiles “total rewards” and the “economic and support” in the analysis of Canadian workers [15] and the Flemish “low stress” profile [13]. However, it does not match a profile in the Eurofound analysis, although a group of identical names exists. The Eurofound SR profile shows favourable social and physical working conditions and – in contrast to the SR profile in this study – low discretion and very low earnings, coupled with very low work intensity [14]. Finally, the HF shows a similar profile to the “high flying” identified by Eurofound [14] with favourable ratings on all indicators, very high earnings and some exposure to work intensity. However, while this profile constitutes an exceptional work exposure constellation in this study of workers in Germany (3%), it covers 21% of all participants in the Eurofound survey. Thus, the SR and the HF in the German LPA assessment do not match occupational group profiles identified by Eurofound to the same degree as the other profiles do.

### European vs. national profiles

The overall high correspondence of the profile pattern in this study with that from Eurofound may not be surprising as the two studies are quite similar in the conceptual understanding of work and work exposure. They also share similarities in the assessment, i.e. the work indicators selected, their underlying items and, finally, the predominant application of continuous scales in the analysis. The differences between the findings of the two studies may have a simple explanation: The Eurofound analysis is based on a Europe-wide sample with many work quality indices exhibiting a European North-West / South-East gradient in the expected direction [14], which is reflected by the profile distribution. The Eurofound profiles *high flying*, for example, is highly prevalent in North-West Europe (e.g. 39% of all workers in Denmark) and rare in the South-East (e.g. 5% in Greece); similarly the *under pressure* profile. In contrast, the Eurofound profile *smooth running* is characteristic for some central and south European countries with comparably low income (e.g. Bulgaria 47%). Their *poor quality* profile is characteristic for South-East Europe (e.g. 54% in Romania) but not for North-West Europe (e.g. 5% in Finland). Certainly, this uneven distribution of work quality across Europe will influence potential profiles obtained as it may generate characteristic profiles for work in certain economic regions, thereby enabling a European perspective, for example for European social policy. When it comes to the national point of view, however, profiles based on national representative samples are more appropriate to reasonably reflect the target group of interest.

### Relation of profiles to the work-related outcomes

By linking profile assignment to external outcomes, the distinctness of the profiles was confirmed due to observable differences across all of them. For all four work-related outcomes, significant differences were found between the profiles. PQ group membership was associated with adversity with respect to all work-related outcomes considered in this study, indicating a genuine risk group for continued work and employment. At the other extreme, there is no occupational profile with exclusively favourable outcome manifestations. According to their ratings, the HF are markedly physically fit accomplishers, but medium level scorings for mental health and work-privacy-conflict indicate that this group does not constitute an overall favourable group. This is in line with the findings presented by Eurofound for the equivalent HF profile [14]. The large SR profile indicates a group with high resources: good mental health and second best in all other outcomes. Again, this profile finds its equivalent in the Eurofound profile with same name, but with poor work-life balance [14]. The profile with lowest work-privacy-conflict identified in this study is the RM which also exhibits good mental health. This is a group with a favourable social work environment and markedly lowest work intensity. However, the mean score for physical health is low, which may be assumed to substantially reflect working-life-long physical work exposure. It is notable that the two groups with low mean scores for the social work environment indicators show most adverse scores for mental health, work-privacy-conflict and also work ability. Unfortunately, Eurofound does not differentiate between mental and physical health. This, in turn, was done by Vanroelen et al. [13], displaying the profile main effects for emotional problems and musculoskeletal complaints. Yet, the profiles follow widely the same ranking for both outcomes with the low stress profile exhibiting the most and the high stress the least favourable effects.

When observing time effects, a mean deterioration of physical health over seven years was found. With respect to mental health, an unexpected dip in the 2014 assessment was observed for most profiles which may partly be attributed to the fact that the wave 2 assessment was predominantly performed in late winter. Work ability deteriorated strongly, especially for the groups with initially high work ability. With respect to work-privacy-conflict all groups converged over time. The profile*time interactions were significant for all outcomes except physical health, but always with low effect size. Thus, in this study no indications for profile membership were found which at one point in time clearly predict change of work-related outcomes in the future. However, it cannot be ruled out that this will be the case in future assessments when the workers’ age increases and thereby also risks for health and work ability. Limitation of space and aims of this publication do not allow for a deeper discussion of the profile*time interactions with respect to the work-related outcomes.

What has not been considered in the analyses yet is the frequency and the preventive potential of the occupational change in the sample. Some workers may find themselves in *job lock* or *stuck at work* situations [39] not being able to modify their adverse work situation. Others, however, might benefit from occupational change, which comprises change of profession, employer or tasks. Bujacz et al. [8] have found systematic change over a six-year period mainly from more demanding to lesser demanding clusters characterised by psychosocial work exposure. Membership in supportive clusters was found to be fairly stable.

### Translation into policy needs

The originality and strength of the person-centred, typological approach in occupational epidemiology primarily lies in its preoccupation with the social distribution of work exposures and its structuring [13]. This study identified common ways in which older employees in Germany characterise their perception of work. These common perceptions are reflected by the five profiles identified in this study, which now may be considered typical for the older socially insured workers in this country. This profile pattern has some contextual overlap with that of other studies [13, 14]. Yet, it also shows individual features which may in part be explained by the fact that - in contrast to further studies - a rather homogenous sample has been investigated, namely socially insured older workers in one country. The findings also show that profile membership is reflected by substantial and plausible differences on four work-related outcomes. Thus, it may be assumed that “it matters” whether an individual is more likely to fit into the one or the other profile.

Numerous surveys in Germany have been used to identify poor working conditions and their associations with health and wellbeing [40, 41]. In contrast to that, and for the first time in Germany, the findings from this study allow to postulate that working groups exposed to distinctly poor-quality or high-quality work-situations exist, and that these can be quantified and characterised. Accordingly, one third of all older workers in Germany work under predominantly poor working conditions, the PQ and SnM, clearly associated with poor work-related outcomes. At the other extreme, another third of workers finds itself in profiles indicating rather favourable overall working situations (SR and HF), mostly positively related to health, work ability and work-privacy-conflict. Finally, there is one group which covers the remaining 30% of older workers, the RM: Here, both adverse and favourable exposures are found and work-related outcomes indicate both substantial resources and risks.

The knowledge of the five profiles identified may help to understand the older working population to whom the transition from work to retirement becomes increasingly apparent. This transition is not only complex, but also a process encompassing the notion of *leaving* or *staying* in employment [4]. In times of extending working lives, the interest of policy is usually focused on *leaving*, i.e. the timing of retirement. Yet, the findings in this study imply that for some groups of older workers the *staying* may constitute substantial personal challenges. This specifically applies to those 20% of all older workers who belong to the PQ profile, and possibly the SnM, an additional 14%. On the other hand, being aware of the complexity of retirement, one should not assume that membership to a favourable profile such as SR, HF, or RM automatically indicates late retirement, as in Germany an early exit culture still prevails [42].

#### Strengths and limitations

According to the findings in this study, about one third of all workers assessed in this study belongs to one of the two clusters with predominantly less favourable work exposures, the PQ and the SnM. In line with this is the observation that the PQ scores worst on all work-related outcomes assessed from wave 1 throughout wave 3, in most instances followed by the SnM. For a number of reasons this does not necessarily mean that all workers in these profiles were dissatisfied with their work or did not find it meaningful. Firstly, it needs to be considered that when presenting the manifestation of the indices across profiles as in Fig. 1 z-transformed scores are shown. Z-scores are relative scores, indicative of the ranking within the total sample and not of a defined score or cut-off known to represent adverse exposure. However, the accumulation of low z-scores for work quality indices in a profile (such as in PQ) is still indicative of clearly less favourable exposure than that in other profiles. Secondly, while the work-health associations identified for the profiles are mostly in the expected direction and causality seems plausible, we did not investigate this in our study, even if the study is of longitudinal nature. In fact, a reciprocal relationship and even feedback loops are also likely to contribute to the findings: the worker’s health may positively or negatively affect their work, which in return may affect the individual worker i.e. with respect to attitudes, behaviours and health, with the potential for further feedback loops [43]. Finally, some workers may not perceive “poor” work as “poor”, possibly because their work represents what they have been experiencing throughout their working life and which has shaped their expectations. When interpreting the results, one has to keep in mind that the work indices assessed are measured at a cross-section in time, yet, they are assumed to reflect chronic work exposure of relatively enduring character. The stability of the work exposures and thereby profile allocation needs to be assessed in further studies.

Methodically, the stochastic regression imputation chosen to replace missing values mainly on the earnings indicator may have several advantages over more convenient methods like mean imputation. Nonetheless, it is still limited to being a single imputation method and the standard errors of the imputed values tend to be underestimated because an uncertainty component is not included in the prediction of the missing values [27]. Unfortunately, the use of a single data set was necessary for analyses with two statistic programs which prevented application of more advanced imputation methods like multiple imputation. In sum, the chosen imputation method served as a pragmatic trade-off between maximum possible reduction of potential bias due to missing values and technical aspects of the analysis process.

The LPA conducted here is advantageous over deterministic clustering techniques, because posterior profile assignment is probability-based and not based on e.g. distance measures [44]. Here, the degree of dissimilarity of each individual with the identified classes is quantified because the highest probability of belonging to a certain cluster seldom equals 1.0. This represents a more appropriate localisation of the individual work exposure relative to all other workers in the sample under study. Nonetheless, one has to keep in mind that this advantage is obscured by the posterior profile assignment for practicability reasons.

As for the outcome measures chosen to validate the identified profiles, parametric tests were applied to detect differences over the course of three assessments. In ANOVA violations of the underlying assumptions can compromise analyses which is why the outcome distributions were inspected beforehand. Slight deviations from normality were evident but of acceptable extent. Moreover, the respective group sizes were large enough to yield results that can be considered reliable, as the ANOVA is robust when assumptions are violated but minimum group sizes are ensured [45].

## Conclusions

In sum, the findings indicate that a differentiated view on the older work force should be applied when it comes to the work-retirement transition. From a policy perspective, the results imply that the political interest should not be limited to the timing of retirement, but also cover quality of work and personal life during the last working years - specifically in the considerable occupational risk groups identified in this study. Broad evidence from different fields of academic research indicates that current extending working life policies bear substantial risks for increasing social inequalities and current empirical studies confirm this with recent data [46]. Here, monitoring is needed - at an early stage - to identify groups of workers representing losers or winners in the transition process. For this, the five work profiles identified in this study may constitute crucial clusters needed to reliably reflect the over-all work exposure patterns in the older work force in Germany.

Research should continue to characterise these five groups with respect to further outcomes, such as economics, financial and social wellbeing, motivation and employment. Further scientific monitoring of the profiles is needed for the early detection of adverse trends in all respects. Not least, the pathways from work to retirement and the individual and social effects related to them will require substantial scientific attention. Finally, it may be of scientific, social and also organisational interest to investigate frequency and consequences of older workers’ change between the five profile groups identified in this study.

## Data Availability

An anonymised Scientific Use File containing data of the first two waves of the lidA study is available by request for scientific purposes at the Research Data Centre (FDZ) of the German Federal Employment Agency at the Institute for Employment Research, IAB, Nuremberg, Germany (https://fdz.iab.de/de/FDZ_Individual_Data/lidA.aspx). Data from the third wave of the study is not publically available yet due strict social data protection requirements. The publication of an approved Scientific Use File covering all three waves of the lidA study is planned for 2023.

## Abbreviations

AIC: Akaike Information Criterion
BIC: Bayesian Information Criterion
COPSOQ: Copenhagen Psychosocial Questionnaire
ERI: Effort-Reward-Imbalance Questionnaire
HF: High Flying
LCA: Latent Class Analysis
lidA: Leben in der Arbeit
LPA: Latent Profile Analysis
PQ: Poor Quality
RM: Relaxed Manual
SnM: Strained non-Manual
SR: Smooth Running
